# “An End to a Means”: How DNA-End Structure Shapes the Double-Strand Break Repair Process

**DOI:** 10.3389/fmolb.2019.00153

**Published:** 2020-01-10

**Authors:** Almudena Serrano-Benítez, Felipe Cortés-Ledesma, Jose F. Ruiz

**Affiliations:** ^1^Andalusian Center of Molecular Biology and Regenerative Medicine (CABIMER-CSIC-University of Seville-Pablo de Olavide University), Seville, Spain; ^2^Topology and DNA breaks Group, Spanish National Cancer Research Center, Madrid, Spain; ^3^Department of Plant Biochemistry and Molecular Biology, University of Seville, Seville, Spain

**Keywords:** DNA double strand break (DSB), Non-homologous DNA end joining, ATM, DNA-PK catalytic subunit, genome instability

## Abstract

Endogenously-arising DNA double-strand breaks (DSBs) rarely harbor canonical 5′-phosphate, 3′-hydroxyl moieties at the ends, which are, regardless of the pathway used, ultimately required for their repair. Cells are therefore endowed with a wide variety of enzymes that can deal with these chemical and structural variations and guarantee the formation of ligatable termini. An important distinction is whether the ends are directly “unblocked” by specific enzymatic activities without affecting the integrity of the DNA molecule and its sequence, or whether they are “processed” by unspecific nucleases that remove nucleotides from the termini. DNA end structure and configuration, therefore, shape the repair process, its requirements, and, importantly, its final outcome. Thus, the molecular mechanisms that coordinate and integrate the cellular response to blocked DSBs, although still largely unexplored, can be particularly relevant for maintaining genome integrity and avoiding malignant transformation and cancer.

Double-strand breaks (DSBs) are the most devastating lesion that DNA molecule can suffer. Indeed, they can cause dangerous chromosomal rearrangements or even cell death if they are not properly repaired. In general terms, there are two conceptually different pathways to repair DSBs that can be divided into those that use homologous sequences—either a sister chromatid or another sequence elsewhere in the genome—as a template in the repair (homologous recombination, HR), and those that directly rejoin the ends, without any template requirement (Lieber, 2008; San Filippo et al., 2008; Pannunzio et al., 2018), regardless of whether using minimal (non-homologous end-joining, NHEJ) or more extensive (microhomology-mediated end-joining, MMEJ) microhomologies to stabilize the junctions. Despite the general intrinsic diploidy of somatic mammalian cells, HR rarely uses the homologous chromosome as a template for DSB repair (Johnson, 2000). Consequently, HR is mostly restricted to late S/G2 phase, when a sister chromatid is available, whereas NHEJ can operate in any phase of the cell cycle. Besides this global distinction, there are additional peculiarities of DSB repair mechanisms based on the specific nature of each DNA lesion, specifically when it comes to the chemical configuration of the broken DNA ends. In this regard, since the HR will use the information of an intact template for repair (San Filippo et al., 2008), the ends of the break, both 5' and 3′, can be extensively degraded without compromising an efficient reconstitution of the initially lost DNA sequences. In contrast, the chemical modifications of DSB ends, and how these are solved, are pivotal in the NHEJ process and final repair outcome. It is therefore of great interest to understand how DSBs harboring complex DNA ends are repaired in the G1 phase of the cell cycle, during which HR is strongly limited.

## The NHEJ Process

The starting point of the NHEJ process takes place with the recognition and binding of double stranded DNA ends by the KU70/80 heterodimer, which occurs in an extraordinarily efficient manner due to its abundance and its strong avidity for this type of DNA substrate. DNA-bound KU heterodimer, in turn, recruits DNA-PKcs to form the DNA-PK holoenzyme, so that the two DNA-PKcs molecules bound to opposing sides of the DSB can interact one each other, contributing to synapsis of broken DNA ends (Meek et al., 2008; Neal and Meek, 2011). The DNA-PK complex is the main regulator of the NHEJ process, coordinating the recruitment of downstream NHEJ accessory factors, such as X-ray cross complementing group 4 (XRCC4), XRCC4-like factor/Cernunnos (XLF), or Paralog of XRCC4 and XLF (PAXX), and DNA ligase IV (LIG4), which contribute to the proper pairing of DSB ends and perform the final ligation of the break (Kakarougkas and Jeggo, 2014; Ochi et al., 2015; Conlin et al., 2017). In vertebrates, NHEJ further evolved an end processing capacity that allows for the repair of complex ends (e.g., hairpins), and which is also, in part, regulated by DNA-PK, as will be discussed below.

## Relevance of End Structure and Configuration During NHEJ

**Figure 1:**
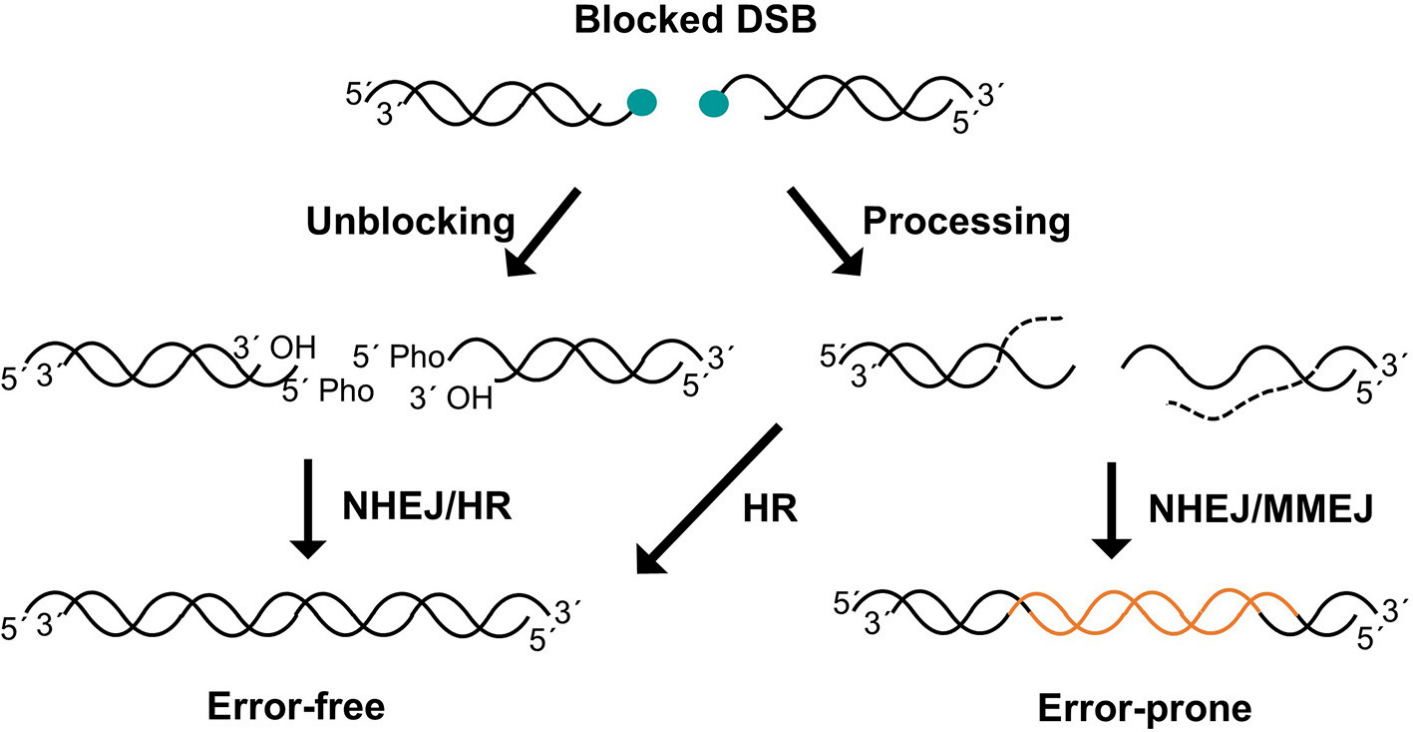
Unblocking and processing of DSBs. Unblocking pathways directly convert ends into 5′-phospahte and 3′-hydroxyl but the nucleotide sequence remains intact, promoting error-free repair (**left**). Processing can also facilitate blocked DSBs repair removing aberrant structures from DNA ends by nucleotide trimming (**right**). This pathway can lead to error-prone repair when non-templated repair pathways such as NHEJ or MMEJ are used. 5′ blocks are depicted but similar situations could be generated on 3′ ends.

**Figure 2:**
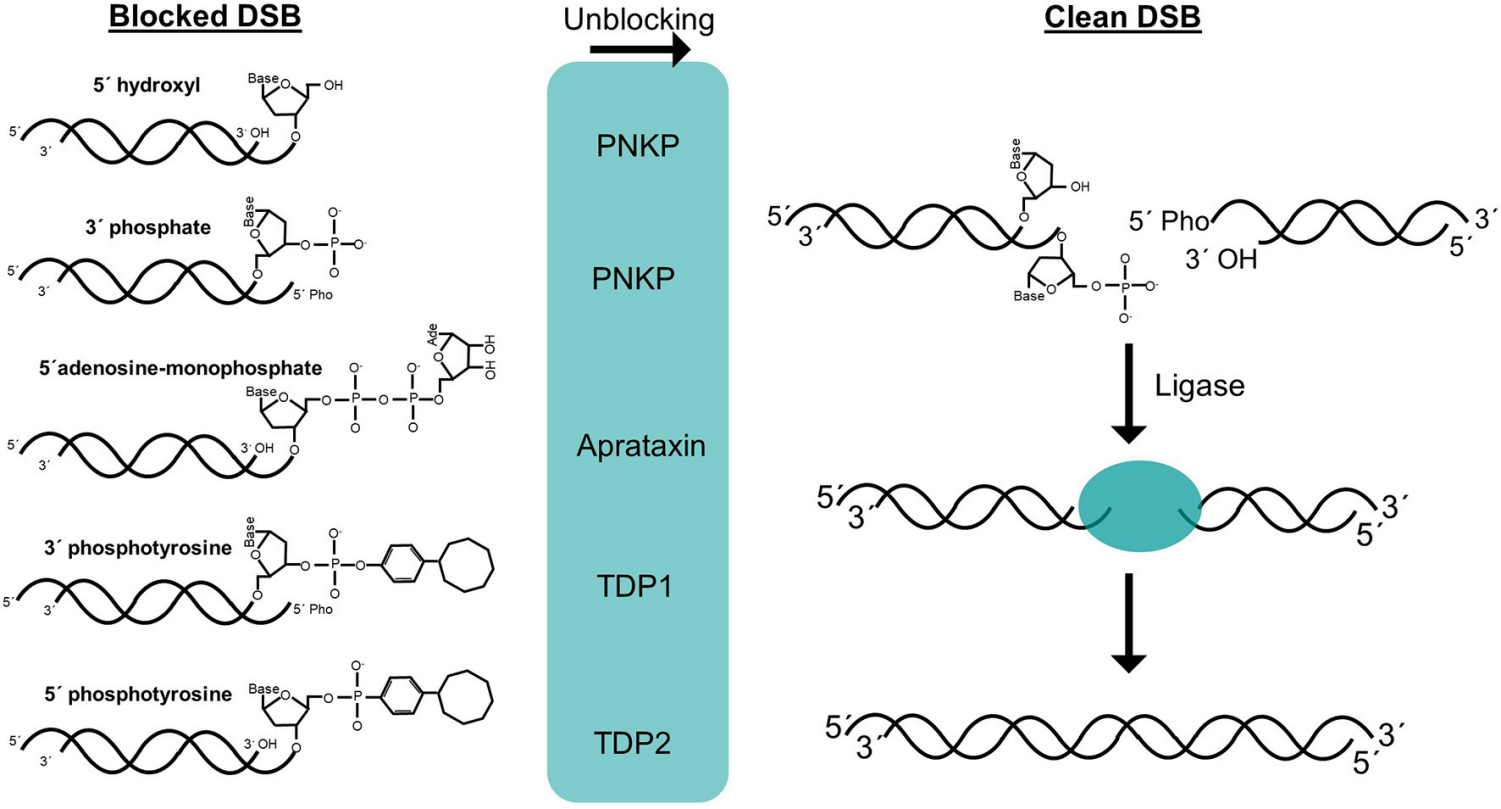
Structure of DNA ends and unblocking enzymes. DSB termini can be blocked by numerous chemical structures *in vivo*. Several unblocking enzymes are present in mammalian cells and efficiently convert these structures to clean 5′-phosphate and 3′-hydroxyl DSB termini (**left**). These clean DSBs can be in theory, directly repaired with the only enzymatic activity of a ligase (**right**).

It can be claimed that the only essential step of NHEJ process is the ligation of one of the DNA strands of the DSB (Waters et al., 2014). During this process, LIG4 activity requires compatible ends harboring canonical 5′-phosphate and 3′-hydroxyl termini. However, DSBs often have complex ends with chemical modifications or structures that do not allow straight-forward joining of the termini, so they can be considered as blocked ends (Figure 1). These chemical variations can be sensed by LIG4 through the disruption of its catalytic cycle (Reid et al., 2017). Therefore, when DSBs harbor non-canonical chemical structures at the ends, they must be restored to conventional 5′-phosphate and 3′-hydroxyl termini so that DNA ligation can take place. There are two conceptually different ways by which these non-canonical DNA ends can be converted into ligatable substrates (Figure 1). On the one hand, cells have a variety of enzymes to directly restore the canonical chemical structure. Given that this event does not involve any sequence modification, it can be simply considered as an “unblocking” process. On the other hand, under certain circumstances, such as the presence of complex lesions, unblocking activities may be compromised or overwhelmed, resulting in DSBs that require additional “end processing” by the action of nucleases that cleave DNA sequence from the ends to remove the chemical modifications (Figure 1). Regarding unblocking, there is a large number of factors with different enzymatic activities that are available for this process during NHEJ (Figure 2), such as tyrosyl-DNA phosphodiesterases 1 and 2 (TDP1 and TDP2, respectively), polynucleotide kinase (PNKP), Aprataxin, and even KU. This, in turn, reflects the wide variety of damaged termini that can arise, as each of these factors removes specific chemical modifications at DNA ends (Povirk, 2012; Andres et al., 2015). These unblocking activities are essential in NHEJ, since they are responsible for facilitating accurate religation of the breaks, as opposed to the processing of DNA ends that may involve nucleotide loss or gain and, therefore, sequence modification. Interestingly, ionizing radiation, which is a common and well-established source of DSBs, mostly induces blocked termini with heterogeneous end structures. Damage occurs either directly, by high-energy particle collision with DNA, or indirectly, when these particles split water molecules leading to dangerous free radicals; in both cases this mainly results in breakage of the sugar backbone, and therefore needs to be processed, necessarily leading to loss of one nucleotide from the termini (Reisz et al., 2014).

Another aspect to highlight in NHEJ is the possible incompatibility among DSBs to be repaired due to the absence of sequence complementarity of DNA ends. This situation may occur when DSB ends have small protuberances, either with 5′ or 3′ polarity. The short stretches of single stranded DNA of these overhangs may be compatible (either fully or partially complementary sequences) or not. It has been shown that LIG4 can ligate across short gaps or rejoin several incompatible DNA end configurations that do not share even 1-bp of terminal microhomology (Gu et al., 2007). For this scenarios, NHEJ also takes advantage of several processing enzymes that can modify DNA ends until they become ligatable substrates (Strande et al., 2012). In this way, single-stranded DNA overhangs (as also may happen with blunt ends) can be trimmed by nucleases such as ARTEMIS giving rise to small gaps than can be efficiently filled-in by specialized X family DNA polymerases (see below) (Mahajan et al., 2002; Lee J. W. et al., 2004; Ma et al., 2004; McElhinny et al., 2005; Capp et al., 2007; Lieber, 2010). It is worth noting that non-complementary DNA ends are indeed the most likely result of end processing at initially chemically modified structures.

## NHEJ: an Iterative Vs a Hierarchical Process

Although NHEJ is generally considered a single DNA repair pathway, a wide variety of factors are needed and different sub-routes can be distinguished depending on the different DSB end configurations (Pannunzio et al., 2018). In addition, there is still important debate about how these NHEJ accessory factors actually operate, and, in this sense, two apparently antagonistic positions can now be distinguished. On the one hand, some authors propose that NHEJ factors operate in an iterative way without an established order (Gu and Lieber, 2008; Lieber, 2008; Gu et al., 2010). This model highlights the flexibility of the NHEJ process and explains the diversity of repair products generated from the same type of DSB. The iterative nature of this process implies that multiple NHEJ components can act on the same DSB during multiple consecutive rounds of processing and that the involvement of factors is not mutually exclusive to the usage of other ones, all of them remaining active as long as the DSB continues unrepaired. On the other hand, other authors propose that there is a hierarchy in NHEJ, by which cells give precedence to resolution paths with the fewest number of enzymatic steps. This way, direct ligation is favored over more complex pathways that include end-processing and/or DNA synthesis (Waters et al., 2014). Consistent with this, LIG4 is the most flexible ligase known so far (Ma et al., 2004; Gu et al., 2007), and the differences in how their catalytic domains interact with different end structures trigger dramatic changes in the dynamics of the entire NHEJ complex, determining the steps taken to complete repair and the factors required (Conlin et al., 2017). A hierarchical order in the action of NHEJ components is also supported by the formation of a synapsis with two different stages (Graham et al., 2016). First, DNA ends are tethered sufficiently far apart, and are then closely aligned by DNA-PK, XLF, and the LIG4-XRCC4 complex. It has been suggested that this structural conversion can be coordinated with end-processing by changes in the phosphorylation profile of DNA-PKcs (Graham et al., 2016), which would provide a mechanism for the regulation of end processing and ligation. Although both models could seem contradictory, they may not be mutually exclusive. While, NHEJ could behave as an iterative process in which various components can be loaded and act in various combinations without an established order, providing flexibility and efficiency to the repair process, the decision of how complex ends are repaired should not be stochastically determined, as specific unblocking activities must be preferred over end-processing in order to avoid sequence modification.

## Nucleases in NHEJ

As mentioned above, under certain circumstances, DSBs require end processing by the action of nucleases. Usually, these nucleases remove chemical modifications and blockages or cleave mismatched ends by trimming 5′ or 3′ termini through exo- and/or endonucleolytic processing to expose short regions of microhomology between strands and promote end joining (Pannunzio et al., 2018). ARTEMIS is the major nuclease implicated in end-processing during NHEJ (Ma et al., 2002; Goodarzi et al., 2006; Yannone et al., 2008). Its main role takes place during V(D)J recombination, where it is responsible for the opening of DNA hairpins formed at coding joints, an endonucleolytic activity that is promoted by phosphorylation in the ABCDE cluster of DNA-PKcs. However, it has been also shown to have DNA-PKcs-independent 5′ exonuclease activity on ssDNA (Pawelczak and Turchi, 2010; Li et al., 2014). Beyond its role in V(D)J recombination, ARTEMIS contribution in NHEJ is still under study, and recent analysis demonstrated that the ARTEMIS-DNA-PKcs complex also promotes the ligation of incompatible overhangs *in vitro* (Chang et al., 2016; Pannunzio et al., 2018). Besides its versatility to act at many different types of DNA ends, there is a common feature in all ARTEMIS substrates: a ss-dsDNA boundary, which is present in a wide variety of different DNA end configurations (Chang et al., 2015; Chang and Lieber, 2016). Interestingly, a novel 3′ endonuclease activity of ARTEMIS has been recently described, that is promoted by XRCC4-LIG4 complex and also independent of DNA-PKcs (Gerodimos et al., 2017). The stimulation of this activity could be as a result of a conformational change due to the interaction with LIG4 (Pannunzio et al., 2018).

Another factor involved in the repair of complex ends requiring end processing is the MRE11 protein from the MRN complex (consisting of MRE11, RAD50, and NBS1). The MRN complex acts as a sensor of DSBs and promotes repair by NHEJ or HR. Specifically, MRE11 exhibits 3′-5′exonuclease and single-stranded and DNA hairpin endonuclease activities (Paull and Gellert, 1998; Trujillo et al., 2003; Lisby et al., 2004; Stracker and Petrini, 2011; Williams et al., 2011). Endonucleolytic cleavage may be of particular importance for DNA ends covalently-bound to Spo11 (Neale et al., 2005), terminated by hairpins (Lobachev et al., 2002) or generated by TOP1 and 2 poisons (Hartsuiker et al., 2009; Quennet et al., 2011; Hoa et al., 2016). Furthermore, recent *in vitro* studies described that NBS1 is essential to promote MRE11 nuclease activities on DNA ends containing protein adducts, while it inhibits MRE11 3′ to 5′ exonuclease degradation of clean ends (Deshpande et al., 2016). Additionally, the function of the MRN complex during resection is stimulated by the phosphorylated form of CTIP (Anand et al., 2016). Remarkably, the nuclease activity of CTIP has been reported to be specifically required for processing complex DSBs, such as those harboring topoisomerase adducts or generated by irradiation. This suggests that the endonuclease activity of CTIP is only necessary for the removal of DNA adducts and not for the resection of unmodified DNA breaks (Makharashvili et al., 2014). This differentiates catalytic and non-catalytic functions of CTIP during end resection, which requirement would be end-structure dependent.

## Polymerases in NHEJ

As mentioned above, as a consequence of the processing of complex DSBs, the participation of other accessory factors such as DNA polymerases of the PolX family is often required. These polymerases are especially suited for filling in the small gaps that are generated when two ssDNA protruding ends with the same polarity and have either none or partial complementarity. The action of the different PolX polymerases during NHEJ seems to be determined by a gradient of template strand dependence after DSB ends are synapsed, with Polλ being completely template-dependent, Polμ having some template requirements and Terminal Deoxynucleotidyl Transferase (TdT) being fully template-independent (McElhinny et al., 2005). Therefore, when 3′-protruding ends at DSBs do not have any complementarity with each other, Polμ and TdT polymerases can add nucleotides for generating *de novo* terminal microhomology at DNA ends (Gu et al., 2007; Davis et al., 2008; Chang et al., 2016). PolX polymerases are recruited to DSBs through the specific interaction between their BRCT domains with NHEJ core factors (Mueller et al., 2008; Boubakour-Azzouz et al., 2012; Malu et al., 2012; Craxton et al., 2018). These interactions favor DSB repair efficiency (Tseng and Tomkinson, 2002; Craxton et al., 2018), and can be facilitated to some extent by DNA-PKcs-mediated phosphorylation (Sastre-Moreno et al., 2017). In fact, systematic analyses to determine how overhang sequence affects the activity of NHEJ polymerases has shown some DNA synthesis patterns that may be coordinated with ligation complex capabilities (Craxton et al., 2018).

## End-Protecting Factors

In addition to all these unblocking and processing factors, other accessory NHEJ components are required to inhibit or restrict degradation of DSB ends, and therefore avoid excessive DNA sequence loss. In this regard, modifications at the chromatin flanking the DSB, such as histone H2AX phosphorylation (Helmink et al., 2011), and the subsequent recruitment of downstream factors of the DNA damage response (DDR), such as MDC1, 53BP1, and BRCA1 (Bekker-Jensen and Mailand, 2010) represent crucial events for the choice of proper repair pathways, regulating to which extent DSB ends are processed. Accordingly, H2AX deficient mice show an increase in genome instability and, in the absence of P53, are prone to tumor development (Celeste et al., 2002, 2003; Bassing et al., 2003). Moreover, in ARTEMIS deficient cells, H2AX was reported to limit the processing of DNA ends by CTIP endonuclease upon induction of blocked DSBs during V(D)J recombination, this function of H2AX being mediated by MDC1 (Helmink et al., 2011). In the same way, 53BP1 has been also shown to regulate end-processing during V(D)J and CSR recombination (Difilippantonio et al., 2008; Bothmer et al., 2010) and to inhibit CTIP-dependent resection in BRCA1 deficient cells at post-replicative stages of cell cycle, suggesting that H2AX phosphorylation may restrict resection by the recruitment of 53BP1 (Bunting et al., 2010). The protective role of DNA ends by 53BP1 requires the participation of some downstream factors, such as PTIP (Kurimasa et al., 2015) and RIF1 (Kienker, 2000; Lee K. J. et al., 2004; Douglas et al., 2005), and maybe other factors yet to be discovered. In this regard, the recently discovered ssDNA-binding complex shieldin has been proposed to act as ultimate effector of the 53BP-RIF1 pathway for end protection (Chan et al., 2002; Ding et al., 2003; Meek et al., 2007). Of note, ARTEMIS was previously identified as a PTIP-binding protein, and, strikingly, as one of main downstream effectors of 53BP1-PTIP pathway (Wang et al., 2014). This suggests that 53BP1 could be promoting limited end-trimming and the repair of DSBs through NHEJ, and therefore directly competing with the HR repair pathway that would entail long resection.

## DNA-PKcs, a Master Regulator of Access to DSB Ends

Despite not being conserved in lower eukaryotes, the activity of this phosphatidylinosytol 3-kinase-related kinase (PI3KK) is a clear requisite for its functioning during NHEJ in mammalian cells (Kienker, 2000; Kurimasa et al., 2015). Although there is a long list of DNA-PKcs substrates, mutational analysis (Lee K. J. et al., 2004; Douglas et al., 2005; Goodarzi et al., 2006; Meek et al., 2008) concludes that DNA-PKcs itself is the only NHEJ factor that has been shown to be a functionally relevant target of its own kinase activity (Chan et al., 2002; Ding et al., 2003; Soubeyrand et al., 2003; Cui et al., 2005; Douglas et al., 2007; Meek et al., 2007, 2008). The most well-accepted consequence of such DNA-PKcs autophosphorylation is its inactivation and dissociation from DNA ends, allowing subsequent joining by LIG4 (Chan and Lees-Miller, 1996; Douglas et al., 2001). Despite the fact that DNA end binding by DNA-PKcs is indifferent to distinct DNA end structures, some studies indicate that cisplatin-DNA adducts near the ends reduce kinase activation, suggesting that free termini could be involved in the activation of DNA-PKcs (Turchi, 2000; Pawelczak et al., 2005). It has been suggested that kinase activation occurs in trans, linking autophosphorylation of DNA-PKcs to synapsis. Although this point is still a matter of debate, this may provide an important mechanism by which DNA-PKcs protects DNA-ends to maintain genomic integrity. However, extensive studies have shown that in response to DSBs, DNA-PKcs autophosphorylation can occur in different residues, with each event having specific functional consequences (Meek et al., 2008; Davis et al., 2014). In human DNA-PKcs, amino acid clusters known as ABCDE, flanking Thr2609 residue, and PQR, around the Ser2056 residue, are the two major phosphorylation sites (Ding et al., 2003; Block et al., 2004; Reddy et al., 2004; Cui et al., 2005; Meek et al., 2007). Although both clusters can be autophosphorylated by DNA-PKcs itself, the ABCDE cluster can be also phosphorylated by ATM or ATR under different cellular stresses (Chen et al., 2007; Meek et al., 2008; Davis et al., 2010). Site-directed mutagenesis analyses and characterization of animal models of DNA-PKcs deficiency (Blunt et al., 1996; Araki et al., 1997; Taccioli et al., 1998; Beamish et al., 2000; Zhang et al., 2011; Danska et al., 2015; Jiang et al., 2015) have revealed that the specific defect resulting from blocking either ABCDE or PQR phosphorylation is DNA end processing deregulation. Both clusters show antagonistic functions, and whereas phosphorylation in the ABCDE cluster promotes DNA end processing, phosphorylation of sites within the PQR cluster inhibits DNA end resection. Specifically, the ABCDE cluster is reported to promote end processing by regulating the access of ARTEMIS to the ends (Ma et al., 2002; Cui et al., 2005; Goodarzi et al., 2006; Yannone et al., 2008). On the other hand, end-ligation requires a strict DNA-PKcs autophosphorylation, possibly in the PQR cluster, which is promoted by ligatable ends and synapsis. This way, possible unsuccessful ligation attempts are avoided. Thus, DNA-PKcs can be considered a molecular shift that coordinates end processing and ligation through its phosphorylation to maximize the efficiency of the NHEJ pathway.

## ATM, a Key Factor to Orchestrate End Processing

Ataxia Telangiectasia Mutated (ATM) kinase is another member of the PI3KK family, recognized by its function as an apical activator of the DDR in response to DSBs (McKinnon, 2004). Interestingly, the structure of ends is a crucial factor which determines the requirement of ATM for the repair of a DSB (Álvarez-Quilón et al., 2014). Specifically, ATM exclusively facilitates the repair of irreversibly blocked TOP2-mediated DSBs, arising by etoposide treatment in TDP2-deficient background (Álvarez-Quilón et al., 2014). Consistent with this, ATM-mediated repair promotes cell survival and the maintenance of genome integrity, avoiding micronuclei and chromosomal aberration formation after the induction of DSBs harboring termini that require end processing (Álvarez-Quilón et al., 2014). Although the underlying molecular mechanisms by which ATM deals with blocked DNA ends are still unclear, two complementary explanations have been proposed (Álvarez-Quilón et al., 2014). On the one hand, ATM can promote limited resection to eliminate the complex structures at DSB ends through the action of nucleases. In this regard, ATM phosphorylates ARTEMIS and DNA-PKcs at the ABCDE cluster (see above) (Chen et al., 2007; Meek et al., 2008; Davis et al., 2010). In addition, a functional interplay between ATM and the MRN complex has been widely reported. Indeed, the three components of the complex are all phosphorylated by ATM, which has been proposed as a modulator of its processing activity (Kijas et al., 2015). Then, the MRN complex interacts with CtIP, which is also positively regulated by ATM to promote end-resection (You and Bailis, 2010; Wang et al., 2013). Finally, ATM regulates other nucleases that could be involved in resolving incompatible ends. This includes APLF (Aprataxin and PNKP-like factor) (Macrae et al., 2008; Fenton et al., 2013); DNA replication helicase/nuclease 2 (DNA2) (Paudyal et al., 2017) or EXO1 (Bolderson et al., 2010; Tomimatsu et al., 2017). On the other hand, ATM could restrict excessive nucleolytic degradation of DNA ends (Rahal et al., 2008). This can actually operate by a direct inhibitory action on aforementioned nucleases such as MRE11 (Rahal et al., 2010) or EXO1 (Bolderson et al., 2010), and/or by promoting modifications at the chromatin flanking the DSB and the recruitment of protecting factors. In this regard, the protective function of H2AX depends on its phosphorylation at Ser139 to form γ-H2AX in chromatin flanking DNA DSBs (Helmink et al., 2011), which is preferentially carried out by ATM (Takahashi et al., 2010). The γ-H2AX downstream factor MDC1 is also phosphorylated by ATM, promoting its oligomerization and spreading on chromatin (Maréchal and Zou, 2013). In addition, ATM phosphorylates 53BP1 (Anderson et al., 2002; Jowsey et al., 2007) and these phosphorylations are required for 53BP1 interaction with PTIP (Munoz et al., 2007) and RIF1 (Chapman et al., 2013). Finally, in addition to these dual end processing/-protective roles, ATM could operate at a later stage in the repair process. For example, after ionizing radiation-induced DSBs, ATM phosphorylates Polλ, which would promote conformational changes in Polλ that facilitate its interaction with NHEJ core factors at DSBs and, hence, stimulates gap-filling DNA synthesis during NHEJ (Sastre-Moreno et al., 2017).

The structure and conformation of DNA ends are therefore determinant to the repair process and outcome, especially in situations in which end-joining mechanisms are prevalent. Although many of the enzymatic activities required have been identified and characterized in detail, the mechanisms by which cells regulate and integrate these activities to keep sequence variation under control are still poorly understood. In this sense, it is tempting to think on blocked DSBs and a deregulated cellular response to these lesions as important threats to genome integrity, and, potentially, drivers of malignant transformation and cancer.

## Author Contributions

AS-B, FC-L, and JR conceived and wrote the manuscript.

### Conflict of Interest

The authors declare that the research was conducted in the absence of any commercial or financial relationships that could be construed as a potential conflict of interest.
